# Supplementary material for: Complex‐centric proteome profiling by SEC‐SWATH‐MS
Source: Mol Syst Biol. 2019 Jan 14;15(1):e8438. doi: 10.15252/msb.20188438 (PMC6346213; doi:10.15252/msb.20188438)
Supplement: Supplementary file 8 — Dataset EV7 [file MSB-15-e8438-s008.zip › feature_plots_string/Q9UQ84.pdf]

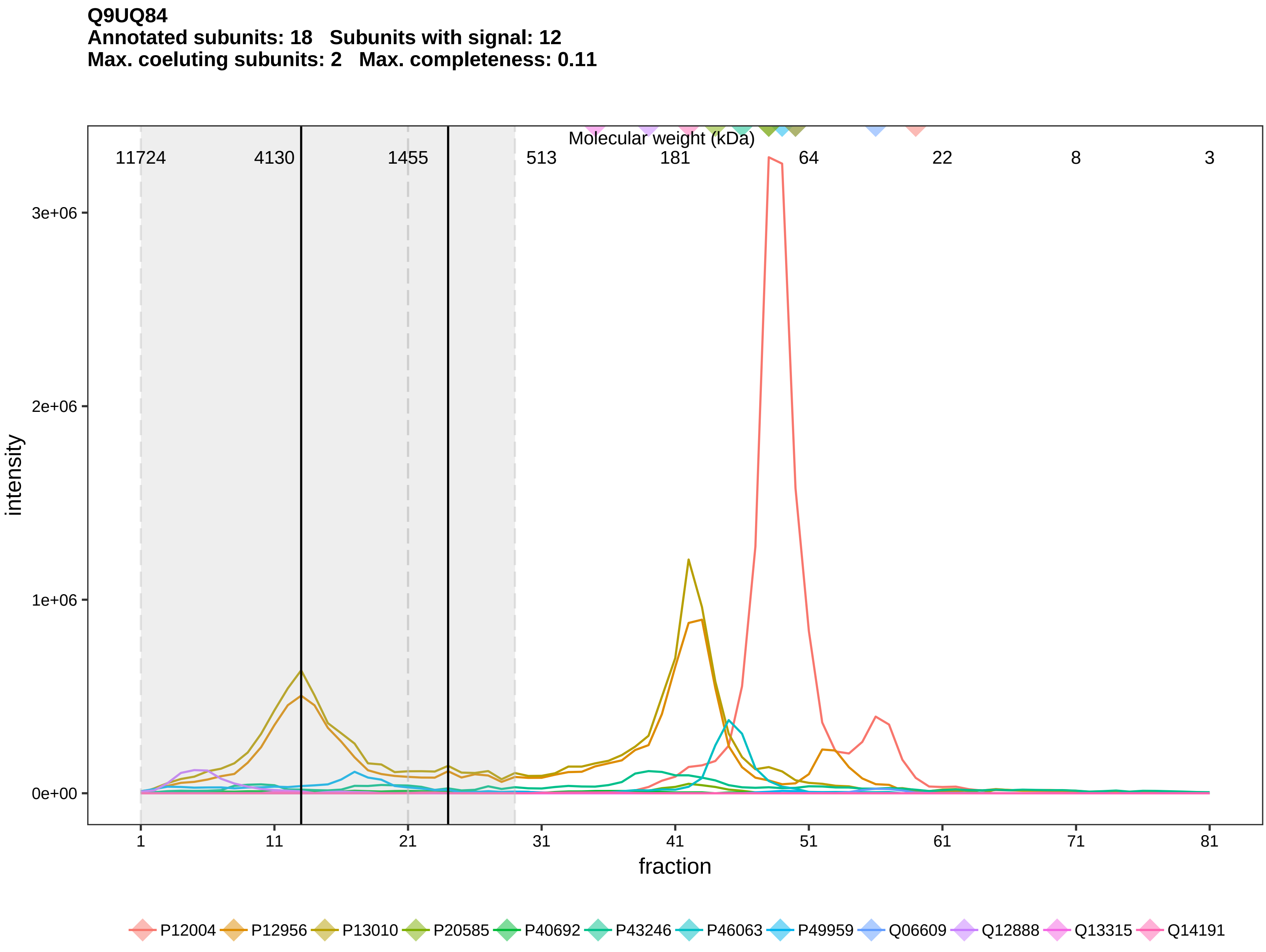
Q9UQ84
Annotated subunits: 18 Subunits with signal: 12
Max. coeluting subunits: 2 Max. completeness: 0.11
Molecular weight (kDa)
11724
4130
1455
513
181
64
22
8
3
intensity
3e+06
2e+06
1e+06
0e+00
fraction
1
11
21
31
41
51
61
71
81
P12004
P12956
P13010
P20585
P40692
P43246
P46063
P49959
Q06609
Q12888
Q13315
Q14191
